# Micronutrient status and risk of *Mycobacterium tuberculosis* infection in Indonesian tuberculosis case contacts

**DOI:** 10.1093/trstmh/trae140

**Published:** 2025-01-16

**Authors:** Ayesha J Verrall, Lisa Houghton, Lika Apriani, Harold E Atmaja, Arjan van Laarhoven, James E Ussher, Rovina Ruslami, Katrina Sharples, Susan McAllister, Reinout van Crevel, Philip C Hill, Bachti Alisjahbana

**Affiliations:** Department of Pathology and Molecular Medicine, University of Otago, Wellington 6021, New Zealand; Department of Human Nutrition, Division of Sciences, University of Otago, Dunedin 9054, New Zealand; Research Center for Care and Control of Infectious Disease, Universitas Padjadjaran, Bandung 45363, Indonesia; Department of Public Health, Division of Epidemiology, Faculty of Medicine, Universitas Padjadjaran, Bandung 45363, Indonesia; Pharmacokynetic Laboratory, Faculty of Medicine, Universitas Padjadjaran, Bandung 45363, Indonesia; Department of Internal Medicine and Radboud Center for Infectious Diseases, Radboudumc, Nijmegen 6525, The Netherlands; Department of Microbiology and Immunology, University of Otago, Dunedin 9054, New Zealand; Research Center for Care and Control of Infectious Disease, Universitas Padjadjaran, Bandung 45363, Indonesia; Department of Biomedical Sciences, Division of Pharmacology and Therapy, Faculty of Medicine, Universitas Padjadjaran, Bandung 45363, Indonesia; Department of Mathematics and Statistics, University of Otago, Dunedin 9054, New Zealand; Centre for International Health, Division of Health Sciences, University of Otago, Dunedin 9054, New Zealand; Department of Internal Medicine and Radboud Center for Infectious Diseases, Radboudumc, Nijmegen 6525, The Netherlands; Centre for International Health, Division of Health Sciences, University of Otago, Dunedin 9054, New Zealand; Research Center for Care and Control of Infectious Disease, Universitas Padjadjaran, Bandung 45363, Indonesia; Department of Internal Medicine, Faculty of Medicine, Universitas Padjadjaran, Hasan Sadikin Hospital, Bandung 40161, Indonesia

**Keywords:** iron, micronutrients, *Mycobacterium tuberculosis* infection

## Abstract

**Background:**

Certain micronutrient levels have been associated with the risk of developing TB disease. We explored the possible association of selected at-risk micronutrient levels with the development of *Mycobacterium tuberculosis (M.tb)* infection.

**Methods:**

This cohort study in Bandung, Indonesia, followed Interferon Gamma Release Assay (IGRA) negative TB case contacts with a repeat IGRA test at 3 mo. At baseline, blood was analysed for haemoglobin, 25-hydroxyvitamin D, retinol-binding protein, C-reactive protein, alpha-1-acid glycoprotein, serum transferrin receptor (sTfR), ferritin, zinc and selenium. Total body iron was calculated using ferritin and sTfR status. Associations between case contact micronutrient concentration and IGRA conversion were estimated using Poisson regression.

**Results:**

Of 430 contacts, 115 (27%) underwent IGRA conversion. Ferritin concentration (adjusted for inflammation) was positively associated with risk of IGRA conversion (incidence rate ratio [IRR] for ferritin=1.17; 95% CI 1.01 to 1.35; p=0.03), but other select micronutrients were not. This association held for ferritin in the final multivariable model (IRR=1.27; 95% CI 1.09 to 1.47; p=0.002).

**Conclusions:**

The risk of developing *M.tb* infection, as defined by IGRA conversion, is associated with increasing ferritin. Interventions in TB case contacts to temporarily reduce iron levels, including considering withholding any iron supplementation, may be worthy of evaluation.

## Introduction

Historic observations suggest susceptibility to TB disease is influenced by host nutrition. For example, an x-ray survey in a German Prisoner of War camp found British prisoners, who received Red Cross rations, had a much lower rate of TB disease than Russians who did not (1.2% vs 19.0%).^1^ An analysis of data from a nutritional and social intervention for children in the preantibiotic era that provided stable employment, housing and nutrition for patients with TB and their families (the Papworth experiment) showed a reduction in TB disease.^2^

There has been very little research on the influence of nutritional status on the development of *Mycobacterium tuberculosis* (*M.tb*) infection after household exposure to a TB case. We previously reported that the risk of a new infection, defined as Interferon Gamma Release Assay (IGRA) conversion, increased 14% with every g/dL increase in haemoglobin at the time of initial screening (95% CI 1 to 30%; p=0.04).^3^ Iron has been shown to be important for the growth of *M.tb*;^4^ an intervention trial of vitamin D supplementation showed a minimal reduction in tuberculin skin test conversion;^5^ and a case control study in Peru reported an increased risk of vitamin A deficiency and TB disease.^6^ However, evidence of the impact of giving vitamin A or other micronutrients on the risk of developing *M.tb* infection is scarce.

In the present study, we therefore investigated whether any of the five micronutrients (iron, zinc, selenium, vitamin A and vitamin D) were associated with the risk of developing *M.tb* infection.

## Methods

### Study design and procedures

This cohort study followed IGRA-negative TB case contacts, aged over 5 y, of sputum smear positive TB patients in the ‘INFECT’ study platform in Bandung, Indonesia, as previously described.^3^ Index case characteristics were recorded and their *M.tb* isolates obtained. Contacts’ demographic characteristics, smoking status, medical history, symptoms and measures of exposure to respective index cases were recorded. Physical examination included assessment of a Bacillus Calmette-Guerin (BCG) vaccination scar, height and weight. Contacts underwent a QuantiFERON-TB Gold In-Tube IGRA test for *M.tb* infection, interpreted according to the manufacturer's instructions. Contacts who were IGRA negative at baseline had a repeat IGRA at 14 wk, whereupon they were labelled as IGRA ‘converters’ or ‘persistently negative’. Associations of anthropometric results with IGRA conversion have been reported previously.^3^

Blood was drawn into a trace element-free evacuated tube (Becton Dickinson) following the International Zinc Nutrition Consultative Group procedures.^7^ Time of blood collection and time of the last meal were recorded before immediate transfer of the blood samples to a chilled container (−4°C) for transport to the base laboratory. Here serum was separated using trace element-free techniques,^7^ and aliquots frozen at −80°C before analysis or shipment on dry ice to the Department of Human Nutrition, University of Otago, New Zealand, for biomarker analysis of zinc, selenium and vitamin D, and to the laboratory of Dr J Erhardt (VitMins Laboratory, Germany) for biomarker analysis of vitamin A retinol-binding protein (RBP), iron and inflammation markers. Haemoglobin was determined by means of a complete blood count using an automated counter (Sysmex XE 5000; Sysmex Corporation).

### Laboratory procedures

Serum vitamin D (as a 25-hydroxyvitamin D) was measured by the validated bioanalytical method^8^ using liquid chromatography-tandem mass spectrometer (LC-MS/MS) at the Pharmocokinetic laboratory, Faculty of Medicine, Universitas Padjadjaran, Bandung. Serum 25-hydroxyvitamin D2 and 25-hydroxyvitamin D3 concentration were determined by isotope-dilution LC-MS/MS.^8^ In brief, 25-hydroxyvitamin D3 and 25-hydroxyvitamin D2 internal standards were reconstituted to working concentrations using spectrophotometry. Frozen serum samples were thawed, denatured with methanol-propanol containing internal standard, vortex-mixed, extracted into hexane and dried under nitrogen. The reconstituted extract was analysed by chromatography on a Base Deactivated Silica C8 High-performance liquid chromatography column, and the metabolites and internal standards were detected by electrospray ionization MS/MS in multiple-reaction monitoring mode on a Waters instrument. Each batch contained four UTAK controls containing known concentrations of 25-hydroxyvitamin D2 and 25-hydroxyvitamin D3 in pooled serum sample (SRM 972, USNIST). The coefficients of variation (CV) for UTAK controls were 4% for 25-hydroxyvitamin D3 medium, 8% for 25-hydroxyvitamin D3 low, 5% for 25-hydroxyvitamin D2 medium and 11% for 25-hydroxyvitamin D2 low.

Serum was analysed in duplicate in the laboratory of Dr J Erhardt (VitMins Laboratory, Germany) for iron biomarkers ferritin and serum transferrin receptor (sTfR), vitamin A biomarker RBP, and inflammation markers C-reactive protein (CRP) and alpha-1-acid glycoprotein (AGP), by a combined sandwich ELISA technique.^9^ Interassay CV of a control sample (n=40) were 2.3% for ferritin, 3.6% for sTfR, 3.6% for RBP, 5.8% for CRP and 8.1% for AGP. Serum zinc and selenium were analysed by inductively coupled plasma MS (ICP-MS) (Agilent 7500ce ICP-MS; Agilent Technologies) at the University of Otago, New Zealand. The CV for a pooled sample (n=20) and control (UTAK, Utak Laboratories Inc.; n=41) for both zinc and selenium were 1.1% and 2.3%, respectively, with the mean results for the UTAK control both within 3% of certified values.

### Statistical analysis

Descriptive statistics (median and IQR or number and %) were used to describe index case and contact demographic and clinical characteristics, anthropometric measures and biomarker concentrations. Zinc values were adjusted for time since last meal, time of sample collection and time from collection to separation (in keeping with evidence of the sensitivity of zinc levels to these factors),^10^ using a linear regression model with ln(Zn) as the dependent variable and logged time variables as independent variables. Following this, serum ferritin, sTfR, selenium, zinc and RBP values were adjusted for the confounding effects of inflammation (with CRP and AGP), using the Biomarkers Reflecting Inflammation and Nutritional Determinants of Anemia regression approach.^11^ Briefly, biomarkers were transformed to meet the assumptions for linear regression (log_e_ all except ferritin, where a square root transformation was used). A linear regression model with the transformed biomarker as the dependent variable and ln(CRP) and ln(AGP) as the independent variables was used.^12^ The regression coefficients of ln(CRP) and ln(AGP) were then used to calculate adjusted values of the biomarkers using a reference concentration (maximum of lowest decile) for serum CRP and AGP to avoid overadjusting the micronutrient biomarkers among individuals with low levels of inflammation. Total body iron (TBI), expressed as mg/kg body weight, was calculated using the logarithm of the ratio of adjusted sTfR to adjusted serum ferritin (SF) as follows: -[log(sTfR/SF)–2.28229]/0.1207.^13^

Means and 95% CIs were calculated, where appropriate, for vitamin D and adjusted concentrations for serum ferritin, sTfR, RBP, TBI, zinc and selenium.

The thresholds for defining suboptimal biomarkers were as follows (using measures adjusted for inflammation): serum ferritin <12 μg/l;^14^ sTfR >8.3 mg/l;^9^ RBP <0.83 μmol/l;^15^ serum zinc <9.9 μmol/l;^16^ and serum selenium ≤0.82 μmol/l;^17^ 25-hydroxyvitamin D <50 nmol/l;^18^ TBI deficiency <0 mg/kg. Inflammation was assessed by serum CRP >5 mg/L and AGP >1 g/L, respectively.^19^

Associations between case contact micronutrient concentration and IGRA conversion were estimated as relative risks using Poisson regression generalised estimating equations, clustering on case.^20^ Robust standard errors account for the clustering in the data and the non-Poisson errors. Observations for each micronutrient variable were scaled by dividing by the overall SD for that variable. The form of the relationships were first examined using fractional polynomial models.^21^ Separate multivariate models were then fit for all the three measures of iron status (ferritin, sTfR and TBI). Only the iron biomarker that explained the highest amount of the variance (*R*^2^) with IGRA conversion was included in the final multivariate model to avoid issues with multicollinearity. Initial multivariable models included known non-nutritional potential confounders (age and gender of index case and contacts, BCG vaccination, body mass index [BMI], smoking, diabetes), as well as a score reflecting degree of exposure to TB. The score was the predicted value from logistic regression of *M.tb* exposure variables (index case: sputum smear grade, cavities, extent of radiographic disease; contacts: hours spent with, and sleeping proximity to, the case) against IGRA results. Model selection was performed using modified backwards selection, with age and gender of case and contact retained.

All analyses were carried out using Stata version 17 (StataCorp LLC. College Station, TX). p<0.05 (two-sided) indicated statistical significance.

## Results

Of 433 contacts recruited to the cohort with a negative IGRA at baseline,^3^ 430 had a complete set of micronutrient measurements and 115 (27%) underwent IGRA conversion. As expected, IGRA converters were likely to be contacts of more infectious TB cases (by cough duration, sputum smear grade and advanced x-ray changes) and tended to have more exposure to their respective index case and be BCG unvaccinated (Table 1). IGRA converters had higher mean haemoglobin concentrations than persistently IGRA-negative contacts. The two groups were otherwise reasonably well balanced by age, gender, ethnicity, smoking and diabetes status.

**Table 1.** Characteristics of 430 household contacts and their 223 linked index^a^ TB cases

	IGRA converter	Persistently IGRA negative	Total
	n=115	n=315	n=430
**Index case characteristics**			
Age, median (IQR), y	38 (28–51)	42 (31–53)	41 (31–53)
Gender:			
Female	59 (51.3)	159 (50.5)	218 (50.7)
Male	56 (48.7)	156 (49.5)	212 (49.3)
Cough duration, median (IQR), d	60 (30–90)	30 (30–90)	30 (30–90)
<2 wk	11 (9.6)	53 (16.8)	64 (14.9)
2 wk–2 mo	67 (58.3)	173 (54.9)	240 (5.8)
>2 mo	37 (32.2)	89 (28.3)	126 (29.3)
Highest smear grade:			
Scanty and 1+	26 (22.6)	131 (41.6)	157 (36.5)
2+	28 (24.4)	81 (25.7)	109 (25.4)
3+	61 (53.0)	103 (32.7)	164 (38.1)
Cavities on x-ray^b^	56 (50.0)	126 (42.1)	182 (44.3)
Extent x-ray^b^ changes, median (IQR)	40 (25–53)	35 (20–60)	40 (25–55)
**Contact characteristics**			
Hours spent with index case, median (IQR)	5 (3–8)	3 (1–7)	4 (1–8)
Sleep proximity to index case:			
Different room	93 (80.9)	251 (79.7)	344 (80.0)
Same room	22 (19.1)	64 (20.3)	86 (20.0)
Age, median (IQR), y	23 (15–36)	22 (12–41)	23 (12–39)
Gender:			
Female	59 (51.3)	168 (53.3)	227 (52.8)
Male	56 (48.7)	147 (46.7)	203 (47.2)
Ethnicity:			
Sunda	103 (89.6)	289 (91.7)	392 (91.2)
Other	12 (10.4)	26 (8.3)	38 (8.8)
BCG vaccinated	85 (73.9)	275 (87.3)	360 (83.7)
Smoking history:			
Never	74 (64.9)	224 (71.1)	298 (69.5)
Current	37 (32.5)	76 (24.1)	113 (26.3)
Quit >6 mo ago	3 (2.6)	15 (4.8)	18 (4.2)
Diabetes^c^:			
No diabetes	102 (88.7)	290 (92.0)	392 (91.1)
Prediabetes	7 (6.1)	14 (4.4)	21 (4.9)
Diabetes	6 (5.2)	11 (3.5)	17 (4.0)
Body mass index, median (IQR), kg/m^2^	21 (18–25)	20 (17–24)	20 (17–25)
Haemoglobin, mean (SD), g/dL	14.1 (1.9)	13.8 (1.6)	13.9 (1.7)

Abbreviations: BCG: Bacillus Calmette–Guérin; IGRA: interferon gamma release assay; RCBG: random capillary blood glucose.

Figures are n (%) unless otherwise indicated.

a Index cases may be represented multiple times if they have more than one contact.

b x-ray available for detailed reading in index cases of 756 baseline IGRA-positive contacts (96.9%), 113 (97.4%) converters and 301 (95.0%) persistently IGRA-negative contacts.

c Diabetes: no diabetes: RCBG<101 mg/dL or HbA1c<5.7%; prediabetes: HbA1c 5.7%–6.4%; diabetes: HbA1c≥6.5%.

In the overall sample population, the prevalences of selenium deficiency and iron deficiency anaemia were low (0.7% and 3.0%, respectively), the prevalence of sTfR was fairly low (6.3%), but prevalences of zinc deficiency and vitamin D deficiency were high (63.7% and 42.2%, respectively) (Table 2). There was no statistically significant difference by IGRA conversion status in the prevalence of deficiency in any of the micronutrient measures (Table 3).

**Table 2.** Proportion of TB case contacts with micronutrient deficiency at baseline by follow-up IGRA status

	IGRA converter (n=115) N (%)	Persistently IGRA negative(n=315) N (%)	Total N (%)	p
Ferritin <12 μg/l	7 (6.1)	39 (12.2)	46 (10.7%)	0.06
Iron deficiency anaemia^a^	3 (2.6)	10 (3.2)	13 (3.0)	0.76
Soluble transferrin receptor>8.3 mg/L	5 (4.3)	22 (7.0)	27 (6.3)	0.32
Total body iron<0 mg/kg	7 (6.1)	33 (10.5)	40 (9.3)	0.17
Retinol-binding protein<0.83 μmol/L	14 (12.2)	38 (12.1)	52 (12.1)	0.98
Zinc^b^<9.9 μmol/L	70 (65.4)	188 (63.1)	258 (63.7)	0.67
Selenium≤0.82 μmol/L	1 (0.9)	2 (0.6)	3 (0.7)	0.80
25-OH vitamin D<50 nmol/L	48 (41.7)	133 (42.4)	181 (42.2)	0.91

Abbreviation: IGRA: interferon gamma release assay.

Data were adjusted for inflammation before categorisation.

a Hb<120 g/L for females and children or <130 g/L for men, and ferritin<12 μg/L.

b Time since last meal was unknown for 25 participants, so adjusted zinc values were known for only n=405.

**Table 3.** Micronutrient biomarker* concentration by follow-up IGRA status

	IGRA converters (n=115)		Persistently IGRA negative (n=315)	
	Mean (SD)	95% CI	Mean (SD)	95% CI
Ferritin (ug/L)	73.2 (48.3)	60.9 to 77.5	62.4 (47.4)	54.0 to 63.3
Soluble transferrin receptor (mg/L)	5.55 (1.78)	5.07 to 5.71	5.76 (1.90)	5.42 to 5.82
Total body iron (mg/kg)	6.65 (4.59)	6.03 to 7.51	5.79 (4.36)	5.48 to 6.35
Zinc (μmol/L)	9.64 (1.05)	9.71 to 10.2	9.69 (1.64)	9.80 to 10.2
Selenium (μmol/L)	1.17 (0.14)	1.14 to 1.19	1.14 (0.14)	1.13 to 1.16
Retinol-binding protein (umol/L)	1.40 (0.62)	1.27 to 1.50	1.42 (0.63)	1.34 to 1.48
25-hydroxyvitamin D (vitamin D) (nmol/L)	54.9 (20.9)	51.0 to 58.7	56.0 (21.9)	53.6 to 58.5

Abbreviation: IGRA: interferon gamma release assay.

*Ferritin, soluble transferin receptor, retinol-binding protein, total body iron and selenium adjusted for inflammation, and zinc adjusted for time of collection and inflammation.

In a univariable analysis that accounted for clustering by index case, there was a statistically significant positive association between ferritin concentration (adjusted for inflammation) and risk of IGRA conversion. The incidence rate ratio (IRR) for scaled ferritin was 1.17, a 17% increase in risk of converting with an increase of 1 SD of ferritin (95% CI 1.01 to 1.35; p=0.03). TBI was also positively associated with IGRA conversion, but was significant only at the α=0.1 level; there were no other statistically significant associations (Table 4). The multivariable analysis with all micronutrient variables included only 386 participants due to missing values for time since last meal (used to adjust zinc measure) and exposure score. The IRR for scaled ferritin was 1.19, but this was no longer statistically significant (p=0.09) (Table 4). There was no evidence of associations between other micronutrient variables and IGRA conversion. A parsimonious model gave a final estimate of the association between scaled ferritin and risk of IGRA conversion as IRR=1.27, an increase of 27% with an increase of 1 SD in ferritin concentration (95% CI 1.09 to 1.47; p=0.002).

**Table 4.** Univariate and multivariate associations between micronutrient biomarkers* concentration by follow-up IGRA status

Micronutrients	IGRA converters(n=115)	Persistently IGRA negative (n=315)	Unadjusted			Adjusted		
Scaled by SD	Mean (SD)	Mean (SD)	IRR	95% CI	p	IRR Main model^a^	95% CI	p
Ferritin (ug/L), scaled	1.53 (1.01)	1.31 (0.99)	1.17	1.01 to 1.35	0.03	1.19	0.97 to 1.46	0.09
Zinc (μmol/L), scaled	6.40 (0.70)	6.43 (1.09)	0.98	0.85 to 1.13	0.77	0.94	0.80 to 1.11	0.46
Selenium (μmol/L), scaled	8.16 (1.00)	7.98 (1.00)	1.13	0.97 to 1.32	0.11	1.05	0.87 to 1.26	0.61
RBP (umol/L), scaled	2.23 (1.00)	2.26 (1.00)	0.98	0.82 to 1.17	0.81	0.87	0.68 to 1.12	0.27
25-hydroxyvitamin D (vitamin D) (nmol/L), scaled	2.54 (0.97)	2.60 (1.01)	0.96	0.83 to 1.12	0.61	0.94	0.80 to 1.11	0.50
						**Model 2** **^b^**	**95% CI**	**p**
Soluble transferrin receptor (mg/L)	2.97 (0.95)	3.08 (1.02)	0.91	0.76 to 1.09	0.32	0.97	0.82 to 1.14	0.68
						**Model 3** **^c^**	**95% CI**	
Total body iron (mg/kg)	1.50 (1.04)	1.31 (0.98)	1.17	0.96 to 1.42	0.12	1.12	0.92 to 1.36	0.25
						**Model 4** **^d^**		
Ferritin (ug/L)						1.27	1.09 to 1.47	0.002

Abbreviations: BCG: Bacillus Calmette-Guerin; BMI, body mass index; IGRA: interferon gamma release assay; IRR: incidence rate ratio; RBP, retinol-binding protein.

*Ferritin, soluble transferin receptor, RBP, total body iron and selenium adjusted for inflammation, and zinc adjusted for time of collection and inflammation and scaled for comparability by dividing observations for each measure by the SD of that variable.

a Main model using ferritin and micronutrients (Zn, Se, RBP, vitamin D) and non-nutritional variables: index case: age, gender and smear grade; contact: age, gender, BCG vaccination, smoking, BMI and exposure score. Excludes 44 observations due to missing values for adjusted zinc (n=25), exposure score (n=19) and vitamin D (n=1).

b Model using soluble transferrin receptor and micronutrients (Zn, Se, RBP, Vit V) and non-nutritional variables: index case: age, gender and smear grade; contact: age, gender, BCG vaccination, smoking, BMI and hours spent with index case. Excludes 44 observations due to missing values.

c Model using total body iron and micronutrients (Zn, Se, RBP, Vit V) and non-nutritional variables: index case: age, gender and smear grade; contact: age, gender, BCG vaccination, smoking, BMI and hours spent with index case. Excludes 44 observations due to missing values.

d Final parsimonious model includes ferritin, age of contact, BCG, BMI and exposure score. Excludes 19 observations due to missing values for exposure score (n=19).

## Discussion

To the best of our knowledge, this is the first study investigating multiple micronutrient status and risk of developing *M.tb* infection. Our findings show that, in addition to the previously identified association of increasing haemoglobin with IGRA conversion, there is also a positive association for ferritin. There were no associations found between zinc, selenium, vitamin A or vitamin D status and IGRA conversion.

The association of increasing ferritin with IGRA conversion is consistent with studies that have demonstrated iron is a nutritional requirement of *M.tb* infection.^4^ We also note that, with respect to TB disease, the iron transporter natural resistance-associated macrophage protein (NRAMP) is strongly associated with susceptibility.^22^ In a Tanzanian study, high plasma ferritin was associated with increased treatment failure in TB cases and low ferritin levels were associated with increased mortality.^23^ Increasing hepcidin, a regulator of iron homeostasis, levels correlated with increasing time to culture negativity in Japanese TB cases.^24^ Together, these findings indicate that increased availability of iron increases the capacity of *M.tb* to cause infection as well as disease, consistent with the pathogen's nutritional dependency on iron.

We found no association with IGRA conversion for the remaining micronutrients investigated. However, there is some reason to believe that other micronutrient supplementation may have a role in relation to both *M.tb* infection and disease, at least in certain situations. An intervention trial in vitamin D-deficient children in Mongolia showed a borderline significant reduction in tuberculin skin test conversion over 6 mo: five (11%) children receiving vitamin D supplementation underwent conversion compared with 11 (27%) receiving placebo (RR 0.41; 95% CI 0.16 to 1.09; p=0.06).^5^ Vitamin A and selenium deficiencies have been associated with progression to TB disease^6,25^ likely mediated through their effects on antimycobacterial immunity.^26^ Micronutrient supplementation (vitamins A, B complex, C, and E, as well as selenium) reduced disease recurrence by 45% in TB patients in a randomised trial in Tanzania,^27^ but has not been useful for the treatment of TB patients. Intervention trials have found no benefit of zinc or vitamin A supplementation, or other micronutrient supplementation, on treatment success.^28,29^

This study had some strengths. It was a prospective cohort study of people after exposure to a highly infectious TB case at risk of developing a new *M.tb* infection. IGRA tests were used to define infection so that repeat testing was not subject to a boosting effect. The study was large enough to detect significant associations of clinical relevance, but a larger study may be necessary to detect significant associations between some micronutrients and *M.tb* infection. While deficiency of zinc (64%) and vitamin D (42%) among our sample population were high, selenium (<1%) and vitamin A deficiency (12%) were low.

The study did have some limitations. A relatively large number of household contacts were necessarily excluded from cohort follow-up as they were already IGRA positive due to the current or previous exposure. A pre-exposure measurement is also not possible for a household contact study. As such, IGRA converters had most likely been responding to an infection at the time of the baseline test and the infection itself may have affected their baseline results. If the adjustment of ferritin results by CRP and AGP does not fully account for low-grade inflammation, then this could have affected the validity of the ferritin result in relation to its ability to reflect iron status, as it is known to be an acute phase reactant. However, the result for ferritin is consistent with our previously published haemoglobin finding in this cohort and with the non-significant trend with respect to total body iron.

There are several possibilities for further research. As noted above, a larger study may pick up significant associations with micronutrients other than ferritin. Because *M.tb* infection risk may be increased in case contacts with higher ferritin, TB case contacts may benefit from temporary reductions in iron levels by chelating agents,^30^ a hypothesis that could be tested first in animal models. Studies of pausing any iron supplementation in TB case contacts after a known exposure are also warranted. The success of such initiatives could be followed by exploring whether it would be useful to reduce iron levels more chronically in other populations that are highly exposed to *M.tb*, but this may have other negative impacts such as anaemia and tiredness.

## Data Availability

The data underlying this article will be shared upon reasonable request to the corresponding author.
